# Molecular Epidemiology and Antimicrobial Susceptibility of *Clostridium difficile* Isolates from a University Teaching Hospital in China

**DOI:** 10.3389/fmicb.2016.01621

**Published:** 2016-10-17

**Authors:** Jing-Wei Cheng, Meng Xiao, Timothy Kudinha, Fanrong Kong, Zhi-Peng Xu, Lin-Ying Sun, Li Zhang, Xin Fan, Xiu-Li Xie, Ying-Chun Xu

**Affiliations:** ^1^Department of Clinical Laboratory, Peking Union Medical College Hospital, Chinese Academy of Medical SciencesBeijing, China; ^2^Graduate School, Peking Union Medical College, Chinese Academy of Medical SciencesBeijing, China; ^3^School of Biomedical Sciences, Charles Sturt UniversityOrange, NSW, Australia; ^4^Centre for Infectious Diseases and Microbiology Laboratory Services, Westmead HospitalSydney, NSW, Australia; ^5^Teaching and Research Section of Clinical Laboratory, School of Public Health, Taishan Medical SchoolTaian, China

**Keywords:** *Clostridium difficile*, molecular typing, antimicrobial resistance, surveillance, China

## Abstract

While the developed world has seen a significant increase in the number of scientific articles on *Clostridium difficile* infection (CDI), the developing world still lags behind on this subject due to limited laboratory capacity, low awareness, and limited surveillance of this problem. As such, CDI is considered a neglected but potentially huge problem in developing countries. The major aim of this study was to systemically evaluate the utility of several molecular typing tools for CDI, including their relevance in epidemiological studies in developing countries such as China. A total of 116 non-repetitive toxigenic *C. difficile* isolates from Chinese patients, were studied. The isolates comprised 83 (71.6%) A+B+CDT- isolates, 27 (23.3%) A-B+CDT- isolates, and 6 (5.1%) A+B+CDT+ isolates. Typing methods evaluated included multilocus variable-number tandem-repeat analysis, PCR ribotyping, multilocus sequence typing, and sequencing of *slpA* and *tcdC* genes, which identified 113, 30, 22, 18, and 8 genotypes each and exhibited discriminatory powers of 0.999, 0.916, 0.907, 0.883, and 0.765, respectively. Compared to A+B+ strains, A-B+ strains exhibited higher prevalence of drug resistance to clindamycin, erythromycin, levofloxacin, rifampicin, rifaximin, and tetracycline. Furthermore, drug resistance rates of strains with different PCR ribotypes differed, supporting the importance of molecular typing in management and control of CDI. Based on our earlier suggestion to improve the diagnostic laboratory capacity of CDI in developing countries, setting up efficient surveillance programs complemented by relevant molecular typing methods is warranted.

## Introduction

*Clostridium difficile* is a leading cause of both nosocomial and antibiotic-associated diarrhea. The clinical manifestation of *C. difficile* infection (CDI) ranges from asymptomatic colonization to mild diarrhea to toxic megacolon and fulminant colitis. CDI is now considered a public health threat, with a dramatic rise in the incidence and severity of CDI observed in Europe and North America in the past two decades (Rupnik et al., 2009).

Most CDI cases have largely been attributed to the presence of the hyper-virulent *C. difficile* strain BI/NAP1/027 [restriction endonuclease analysis (REA) group BI, North American pulse-field type 1, PCR ribotype 027; McDonald et al., 2005]. Although ribotype 027 strains have caused major epidemics in North America and Europe, only sporadic cases have been reported in Asia (Collins et al., 2013). The major virulence factors for *C. difficile* are enterotoxin A (TcdA) and cytotoxin B (TcdB) (George et al., 1978). However, some strains that produce a binary toxin called *C. difficile* binary toxin (CDT), and whose role in causing disease is not yet clear, have been described and are associated with increased virulence and recurrence rates (Popoff et al., 1988).

Knowledge of the antimicrobial susceptibility profiles and molecular types of *C. difficile* is an important first step for monitoring and understanding the epidemiology of this organism. A variety of molecular methods have been applied for genotyping of *C. difficile*, including PCR ribotyping, pulsed-field gel electrophoresis (PFGE), multilocus sequence typing (MLST), multilocus variable-number tandem-repeat analysis (MLVA), and sequencing of functional genes such as *slpA* and *tcdC* (Collins et al., 2015). Each of these typing methods has its own merits and disadvantages, and may be applied in different occasions according to the aim and scale of the study (Killgore et al., 2008; Collins et al., 2015).

In China, there are limited studies on the molecular epidemiology and antibiotic susceptibility profiles of *C. difficile*, possibly due to insufficient laboratory diagnostic capacity, low awareness, and lack of high-quality surveillance systems (Collins et al., 2013; Hawkey et al., 2013; Cheng et al., 2015). However, in two previous retrospective studies, PCR ribotype 027 isolates were detected, suggesting that the threat of CDI in China is generally neglected and possibly underestimated (Cheng et al., 2016). To partly address this problem, we previously proposed a glutamate dehydrogenase-based algorithm for improving the clinical laboratory diagnostic capacity for CDI in China (Cheng et al., 2015).

The objective of this study was to evaluate the utility of different molecular typing assays for *C. difficile*, in relation to toxigenicity and antimicrobial susceptibility, using isolates obtained from one hospital in China. The usefulness of the different assays in different situations was considered, to inform decision making for improving the capacity of clinical laboratories in the management of CDI in developing countries like China.

## Materials and Methods

### Ethics

The study was approved by the Human Research Ethics Committee of Peking Union Medical College Hospital (PUMCH) (No. PUMCHBC-C-4). Written informed consents were obtained from patients for use of the samples in research.

### Bacterial Isolates

A total of 116 non-duplicate toxigenic *C. difficile* isolates were recovered from patients with suspected CDI in PUMCH between August 2012 and July 2015. The majority of the isolates (69.0%; 80/116) were from the medical wards, followed by outpatient or emergency department (22.4%; 26/116), surgical department (6.0%; 7/116), and finally intensive care units (2.6%; 3/116).

All specimens were initially tested for toxin A/B using enzyme immunoassay (EIA; VIDAS *C. difficile* Toxin A&B, bioMérieux, Marcy l’Etiole, France) and cultured on selective cycloserine–cefoxitin–fructose agar (CCFA) plates. Typical colonies on CCFA were identified as *C. difficile* by matrix-assisted laser desorption/ionization time-of-flight mass spectrometry (MALDI-TOF MS; Bruker Daltonics GmbH, Bremen, Germany). Only *C. difficile* isolates obtained from stool specimens with positive EIA results, and confirmed by MALDI-TOF MS, were included in the study.

### DNA Extraction, Toxin Gene Detection, and Sequencing of *tcdC* Gene

DNA extraction and subsequent toxin gene detection was carried out as previously described (Cheng et al., 2015). Genotype of *tcdC* gene was determined by comparing the obtained sequences with previous published sequences as described by Curry et al. (2007).

### Molecular Type Assays

Capillary sequencer-based PCR ribotyping was performed as described by Indra et al. (2008), and ribotypes were assigned by querying the results against WEBRIBO database^1^. Novel ribotypes were named as “PUR” plus two Arabic numbers (e.g., PUR01).

Multilocus sequence typing was performed by sequencing seven house-keeping gene loci as previously described by Griffiths et al. (2010), and sequence type (ST) and clades of *C. difficile* isolates were determined by querying on official website^2^.

Multilocus variable-number tandem-repeat analysis was performed using the set of seven loci as proposed by van den Berg et al. (2007). Repeat numbers were analyzed using BioNumerics software v6.5 (Applied Maths, Texas, USA) for cluster analysis.

Sequence typing of the *slpA* gene was performed as described previously by Kato et al. (2005). However, due to lack of a consistent nomenclature for *slpA* genotypes, different *slpA* genotypes and subtypes were defined as per NCBI database GenBank accession entries for *slpA* genotype descriptions (Joost et al., 2009).

### Antimicrobial Susceptibility Testing

Antimicrobial susceptibility testing was performed by the agar dilution method according to Clinical and Laboratory Standards Institute (CLSI) guidelines (document M11-A8; CLSI, 2012). The following 11 antimicrobial agents were used: ciprofloxacin, clindamycin, erythromycin, levofloxacin, meropenem, metronidazole, piperacillin/tazobactam, rifampicin, rifaximin, tetracycline, and vancomycin. Interpretation of testing results was based on CLSI M100-S25 (CLSI, 2015), or according to the criteria suggested by Huang et al. (2009) for the drugs whose breakpoints were not available in CLSI documents, as summarized in Supplementary Table S1. *Bacteroides fragilis* ATCC 25285 was used for quality control.

### Data Analysis

The genetic relationships of the isolates was determined by cluster analysis using the minimum-spanning tree available in the BioNumerics software v 6.5 (Applied Maths).

To compare the discriminatory power of different molecular methods, we used an index of discriminatory power (D) based on Simpson’s index of diversity (Fawley et al., 2015).

Statistical analyses were performed using SPSS software (version 17.0, IBM, New York, NY, USA). The chi-square test was applied to compare categorical variables. The level of statistical significance was defined as *P* < 0.05.

## Results

### Toxigenic Types and *tcdC* Genotype

Of the 116 toxigenic strains studied, 83 (71.6%) were *tcdA*-positive, *tcdB*-positive, and *cdtA/cdtB*-negative (A+B+CDT-), while 27 (23.3%) were *tcdA*-negative, *tcdB*-positive, and *cdtA/cdtB*-negative (A-B+CDT-). The remaining six (5.1%) isolates were *tcdA*-positive, *tcdB*-positive, and *cdtA/cdtB*-positive (A+B+CDT+; **Table 1**).

**Table 1:** Multilocus sequence typing (MLST), ribotype, *slpA*, *tcdC*, and toxin genotypes of the 116 *Clostridium difficile* clinical isolates.

MLST clade	MLST ST	Ribotype	*slpA* genotype	*tcdC* genotype	Toxin gene	No. of isolates
Clade 1	ST2	14	hr-01	sc9	A+B+CDT-	2
				WT	A+B+CDT-	4
			pus1-01	WT	A+B+CDT-	1
		20	hr-01	sc9	A+B+CDT-	1
				WT	A+B+CDT-	1
		PUR02	hr-01	sc9	A+B+CDT-	1
		PUR03	cc12078-01	WT	A+B+CDT-	1
	ST3	1	gr-01	sc3	A+B+CDT-	17
	ST8	2	yok-01	WT	A+B+CDT-	3
		PUR04	yok-01	WT	A+B+CDT-	1
		PUR05	yok-01	WT	A+B+CDT-	4
	ST27	39	sh-01	WT	A+B+CDT-	1
	ST35	46	og39-01	WT	A+B+CDT-	8
	ST42	106	hr-02	WT	A+B+CDT-	5
	ST51	PUR07	yok-01	B^a^	A+B+CDT-	1
	ST54	12	kr-03	sc9	A+B+CDT-	16
				WT	A+B+CDT-	3
	ST55	70	kr-04	sc9	A+B+CDT-	1
		PUR08	kr-03	sc9	A+B+CDT-	1
	ST91	PUR10	ar-01	WT	A+B+CDT-	1
	ST98	PUR11	pus1-01	WT	A+B+CDT-	1
	ST129	PUR13	xr-03	sc9	A+B+CDT-	1
				WT	A+B+CDT-	1
	ST233	PUR14	yok-01	sc9	A+B+CDT-	1
	ST278	PUR15	kr-03	WT	A+B+CDT-	1
	ST286	PUR16	pus2-01	WT	A+B+CDT-	1
	ST289	PUR17	hr-01	sc9	A+B+CDT-	1
	ST333	2	yok-01	WT	A+B+CDT-	1
	ST334	PUR12	gc11-01	WT	A+B+CDT-	1
Clade 2	ST1	27	gc8-03	sc1^a^	A+B+CDT+	2
		PUR01	gc8-03	WT	A+B+CDT+	1
Clade 3	ST5	23	kr-04	puc1^a^	A+B+CDT+	1
		63	j52-01	puc1^a^	A+B+CDT+	2
Clade 4	ST37	17	fr-01	sc7	A-B+CDT-	12
				sc9	A-B+CDT-	1
			fr-06	sc7	A-B+CDT-	3
	ST81	PUR09	fr-01	sc7	A-B+CDT-	11
	ST332	PUR06	hr-01	sc15	A+B+CDT-	1

*^a^tcdC-sc1, tcdC-B, and tcdC-puc1 had 18-bp deletion at position 330–347; tcdC-sc1 had a single-nucleotide deletion at position 117.*

Seven previously described *tcdC* STs were identified, including *tcdC*-0 (40 isolates, 34.5%), *tcdC*-B (1 isolate, 0.86%), *tcdC*-sc1 (2 isolates, 1.7%), *tcdC*-sc3 (17 isolates, 14.7%), *tcdC*-sc7 (26 isolates, 22.4%), *tcdC*-sc9 (26 isolates, 22.4%), and *tcdC*-sc15 (1 isolate, 0.9%). One novel *tcdC* genotype (3 isolates, 2.5%) was also identified, and was identical to sequence JF719680 deposited in the GenBank and named as *tcdC*-puc1. Three genotypes, including *tcdC*-sc1, *tcdC*-B, and the novel genotype *tcdC*-puc1, had an 18-bp deletion at position 330–347, which is characteristic of ribotype 027. In addition, the two *tcdC*-sc1 isolates had a single-nucleotide deletion at position 117.

### MLST

The 116 strains were classified into 22 STs. ST54 was the most common (*n* = 19, 16.4%), followed by ST3 (*n* = 17, 14.7%), ST37 (*n* = 16, 13.8%), ST2 and ST81 (*n* = 11, 9.5% each), whilst other STs were rare (prevalence <8%). Two novel STs, ST333 and ST334, were identified (**Table 1**). The majority of isolates (*n* = 82, 70.1%) belonged to clade 1, followed by clade 4 (*n* = 28, 24.1%), and only three isolates each belonged to clades 2 and 3 (**Table 1**).

### *slpA* Genotypes

By *slpA* sequencing, the 116 isolates were discriminated into 13 major genotypes and 18 subtypes (**Table 1**). The dominant *slpA* genotype was fr-01 (*n* = 24, 20.7%), followed by kr-03 (*n* = 21, 18.1%), gr-01 (*n* = 17, 14.7%), yok-01 and hr-01 (*n* = 11, 9.5% each). Two novel *slpA* subtypes, namely pus1-01 and pus2-01, were identified in the present study.

### Capillary Sequencer-Based PCR Ribotyping

Thirty different PCR ribotypes were identified. The dominant ribotype was 012 (*n* = 19, 16.4%), followed by ribotype 001 (*n* = 17, 14.7%), and ribotype 017 (*n* = 16, 13.8%; **Table 1**). Strains of the hyper-virulent PCR ribotype 027 (*n* = 2, 1.7%) were also detected. Other previously undocumented ribotypes, which we designated PUR01–PUR17, were detected. Of these novel ribotypes, ribotype PUR09 exhibited higher prevalence (*n* = 11, 9.5%) whilst the prevalence of each of the other ribotypes was low (0.9–3.4%; **Table 1**).

### MLVA

**FIGURE 1:**
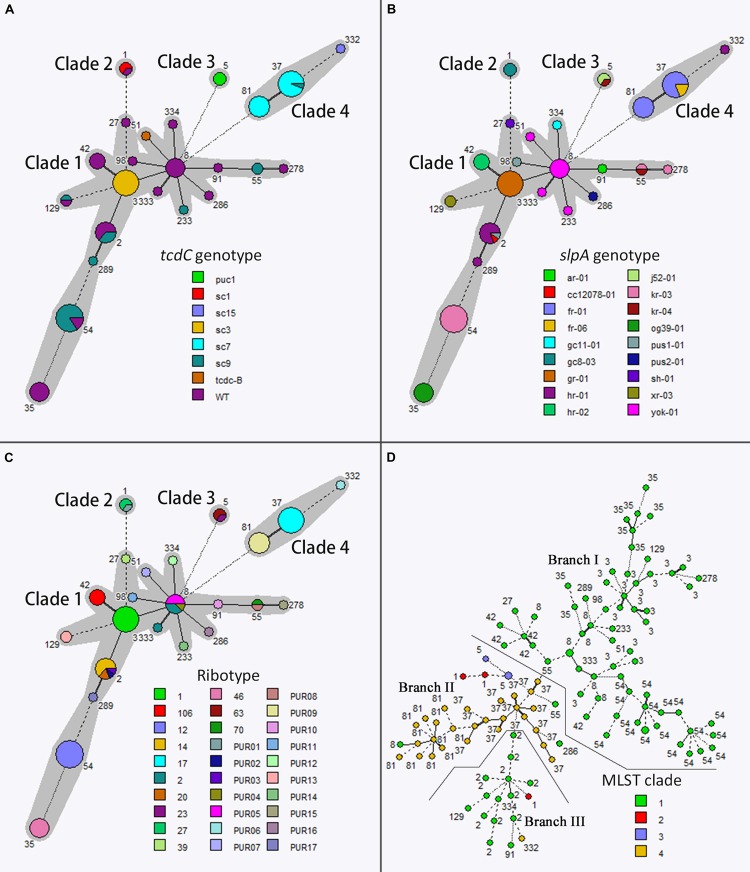
**Minimum spanning tree analysis based on allelic profiles of multilocus sequence typing (MLST) (A,B,C) and Multilocus variable-number tandem-repeat analysis (MLVA) (D) data.** Each circle in **(A,B,C)** corresponds to a MLST ST, and each circle in **(D)** corresponds to a MLVA genotype. Different circle colors represent *tcdC* genotype, *slpA* genotype, PCR ribotype, and MLST clade in **(A,B,C,D)**, respectively. The gray halo surrounding the MLST STs in **(A–C)** denotes STs belonging to different MLST clades. The lines between circles indicate the similarity between profiles: bold line, six of seven MLST alleles/MLVA loci in common; normal line, five alleles/loci in common; dashed line, four alleles/loci in common; dotted line, ≤3 alleles/loci.

Using the seven loci for MLVA typing, namely A6, B7, C6, E7, F3, G8, and H9, we identified 35, 24, 37, 11, 6, 28, and 3 different alleles, amongst 116 isolates, respectively. By combination analysis of the seven loci, a total of 113 different MLVA types were identified (**Figure 1D**). None of the MLVA types identified comprised more than three isolates each.

### Relatedness of Genotypes Determined by Different Typing Methods

Of the different typing methods used, MLVA exhibited the greatest discriminatory power, generating a *D*-value of 0.999, followed by PCR ribotyping (*D*-value 0.916) and MLST (*D*-value 0.907). In comparison to the above, *slpA* and *tcdC* sequencing exhibited lower discriminatory power (*D*-value 0.883 and 0.765, respectively; **Table 2**).

**Table 2:** Discriminatory power of the five typing methods used in this study.

Method	No. of genotypes	Index of discrimination	95% confidence interval
MLVA	113	0.999	0.999–1
Ribotyping	30	0.916	0.895–0.938
MLST	22	0.907	0.888–0.926
*slpA* genotyping	18	0.883	0.858–0.908
*tcdC* genotyping	8	0.765	0.731–0.798

Of note, there was a strong correlation between toxigenic types and MLST clades: all 82 MLST clade 1 isolates were A+B+CDT-. Among 28 MLST clade 4 isolates, 27 (96.4%) were of toxigenic type A-B+CDT- and only one isolate (3.6%) was A+B+CDT-. Furthermore, all A+B+CDT+ isolates were only found in MLST clades 2 and 3 (**Table 1**).

There was also some association between *tcdC* genotyping results and MLST clades: *tcdC*-WT (39/82, 47.6%) and *tcdC*-sc9 (25/82; 30.5%) isolates were the commonest *tcdC* genotype in MLST clade 1, and were rarely represented (one isolate each) in other MLST clades. All the 17 A+B+CDT- isolates which belonged to the second commonest ST in MLST clade 1, i.e., ST3 (17/82, 20.7%), were *tcdC*-sc3. In addition, 96.3% (26/27) A-B+CDT- isolates were *tcdC*-sc7 (**Table 1**; **Figure 1A**).

Of 18 different *slpA* genotypes/subtypes identified, only two were shared by different MLST clades, namely hr-01 shared by MLST clade 1 (*n* = 10) and clade 4 (*n* = 1), and kr-04 shared by MLST clade 1 (*n* = 1) and clade 3 (*n* = 1). Other *slpA* genotypes, although shared by different MLST STs, were unique amongst MLST clades (**Table 1**; **Figure 1B**).

PCR ribotyping exhibited higher discriminatory power than MLST, and none of the PCR ribotypes were shared by different MLST clades (**Figure 1C**). Four ribotypes were identified amongst six A+B+CDT+ isolates. The two ribotype type 027 isolates, which have been previously reported by our group (Cheng et al., 2016), belonged to MLST ST1 (clade 2), *slpA* genotype gc8-03, and *tcdC* genotype *tcdC*-sc1 (having 18-bp deletion at position 330–347, and single-nucleotide deletion at position 117 versus wild-type; **Table 1**). However, the PUR01 (*n* = 1) A+B+CDT+ isolate, which although belonged to MLST ST1 and was of *slpA* genotype gc8-03, carried the wild-type *tcdC* gene (**Table 1**). In contrast, ribotype 63 (*n* = 2) and 23 (*n* = 1) A+B+CDT+ isolates belonged to MLST ST5 (clade 3) and *tcdC* genotype *tcdC*-puc1 (having 18-bp deletion at position 330–347, but with no single-nucleotide deletion at position 117), but were *slpA* genotype j52-01 and kr-04, respectively (**Table 1**). There were only two ribotypes identified in 27 A-B+CDT- isolates, namely ribotype 17 (*n* = 16, 59.3%) and ribotype PUR09 (*n* = 11, 40.7%; **Table 1**). Ribotypes identified in A+B+CDT- isolates were much more diverse (24 ribotypes in 83 isolates), of which ribotype 012 (*n* = 19, 22.9%) and 001 (*n* = 17, 20.5%) were the commonest (**Table 1**).

Multilocus variable-number tandem-repeat analysis exhibited the highest discriminatory power amongst all typing methods used (**Figure 1D**). The minimum spanning tree generated by MLVA also indicated a general phylogenetic relatedness of MLVA typing results and MLST clades (**Figure 1D**). As can be seen clearly in **Figure 1D**, using the MLVA spanning tree, MLST clade 1 was separated into two major branches by MLST clade 4. However, discrepancies between MLVA and MLST were also observed. For example, by MLVA analysis, the three MLST ST1 isolates (red circles in **Figure 1D**) were classified into two different branches: two isolates were more phylogenetically related to MLST clade 3, but one isolate was more closely related to MLST ST2 of MLST clade 1 (**Figure 1D**).

### Antimicrobial Susceptibilities

The minimum inhibitory concentrations (MICs) of 11 antimicrobial agents for the 116 isolates are summarized in **Table 3**. All the isolates were susceptible to metronidazole, vancomycin, meropenem, piperacillin/tazobactam, but resistant to ciprofloxacin. Co-resistance to erythromycin, clindamycin, and levofloxacin was observed in 41.3% of the isolates. High-level resistance to erythromycin (MIC > 128 mg/L) and clindamycin (MIC > 128 mg/L) was detected in 63.8% and 55.2% of the 116 isolates, respectively. The MICs of rifampin were consistent with those of rifaximin, and were either ≤0.064 mg/L or >256 mg/L. Moreover, 25.9% of the isolates were resistant to tetracycline.

**Table 3:** Antibiotic resistance rates and minimum inhibitory concentration (MIC) ranges for the 116 *C. difficile* clinical isolates.

Antimicrobial agent	All strains (*n* = 116)				A+B+ strains (*n* = 89)				A-B+ strains (*n* = 27)				*P*-Value^a^
	MIC_50_ (mg/L)	MIC_90_ (mg/L)	Range (mg/L)	% R	MIC_50_ (mg/L)	MIC_90_ (mg/L)	Range (mg/L)	% R	MIC_50_ (mg/L)	MIC_90_ (mg/L)	Range (mg/L)	% R	
Erythromycin	>256	>256	0.5 – >256	69.0	>256	>256	0.5 – >256	59.6	>256	>256	16 – >256	100	NS
Ciprofloxacin	16	128	8 – 256	100	16	128	8–256	100	128	128	8–256	100	NS
Clindamycin	256	>256	0.25 – >256	87.9	128	>256	0.25 – >256	84.3	>256	>256	32 – >256	100	NS
Levofloxacin	8	256	4 – >256	57.7	4	256	4–256	30.3	256	>256	4 – >256	96.0	<0.001
Meropenem	2	2	0.5–4	0	2	2	0.5–2	0	1	4	1–4	0	NS
Metronidazole	0.25	0.25	≤0.064–1	0	0.25	0.25	≤0.064–1	0	0.125	0.5	≤0.064–1	0	NS
Piperacillin/tazobactam	4/4	8/4	1/4–16/4	0	4/4	8/4	1/4–16/4	0	4/4	8/4	2/4–16/4	0	NS
Rifampicin	≤0.064	256	≤0.064 – >256	12.9	≤0.064	≤0.064	≤0.064 – >256	6.7	≤0.064	>256	≤0.064 – >256	33.3	<0.001
Rifaximin	≤0.064	>256	≤0.064 – >256	12.9	≤0.064	≤0.064	≤0.064 – >256	6.7	≤0.064	>256	≤0.064 – >256	33.3	<0.001
Tetracycline	0.125	32	≤0.064 – 64	25.9	0.125	16	≤0.064–32	12.4	16	64	≤0.064–64	70.4	<0.001
Vancomycin	0.5	0.5	≤0.064 – 2	0	0.5	1	≤0.064–2	0	0.5	0.5	≤0.064–1	0	NS

*NS, not significant.*

*^a^P-Value for resistant A+B+ strains versus A-B+ strains.*

**FIGURE 2:**
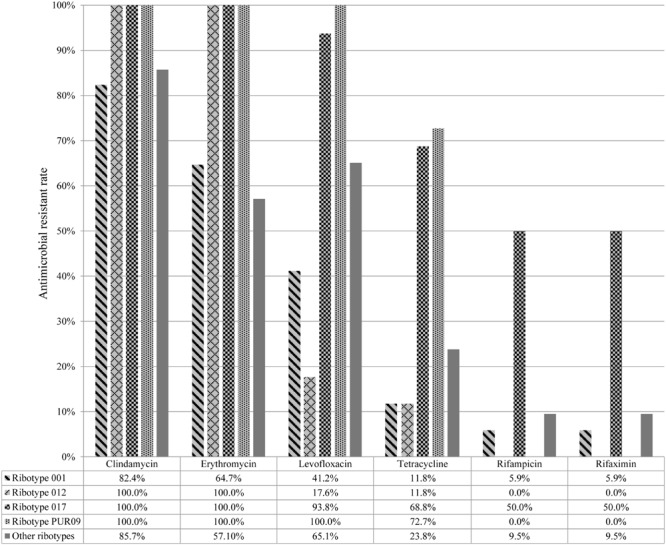
**Drug resistance rates among different PCR ribotypes of 116 *Clostridium difficile* isolates**.

Drug resistance rates for A-B+ isolates for the antibiotics levofloxacin, rifampicin, rifaximin, and tetracycline, were significantly higher than that of A+B+ isolates (*P* < 0.001; **Table 3**). In addition, varied antimicrobial resistance rates were observed amongst different molecular types. For instance, ribotypes 012, 017, and PUR09 isolates exhibited higher resistance rates to clindamycin and erythromycin compared to ribotype 001 and other ribotype isolates (**Figure 2**). Moreover, ribotype 017 and PUR09 isolates showed higher prevalence rate of resistance to levofloxacin and tetracycline, compared to other ribotype isolates. Ribotype 017 isolates also had higher rate of resistance to rifampicin and rifaximin (**Figure 2**).

## Discussion

*Clostridium difficile* infection has become a significant public health threat in the developed world, with substantial increase in morbidity and mortality reported since the early 2000s. However, due to inadequate laboratory diagnostic capacity, little is known about the magnitude of the CDI problem in developing countries like China, and hence the problem may be much underestimated (Collins et al., 2013; Hawkey et al., 2013; Cheng et al., 2016). In this regard, our group has previously attempted to improve the diagnosis of CDI in China by proposing a practical workflow for future CDI laboratory diagnosis (Cheng et al., 2015). Furthermore, the lack of high-quality CDI surveillance systems and epidemiology studies in developing countries contributes to the low awareness of CDI. Thus further studies are needed to address this problem.

Molecular typing methods are essential for understanding the epidemiology of *C. difficile*, and are especially important as part of an active surveillance and hospital infection control strategy for this organism. To date, several typing methods have been used to study the epidemiology of *C. difficile*, and there are pros and cons for each method. Most studies evaluating the utility of different molecular typing methods for CDI are from the developed world. In the present study, we evaluated the performance and utility of the most commonly used genotypic assays for *C. difficile*, including toxigenic typing, *tcdC* genotyping, *slpA* genotyping, MLST, PCR-ribotyping, and MLVA.

Although the dominant *C. difficile* toxigenic type is A+B+, there has been an increase in the prevalence and significance of the A-B+ type in some regions of the world (Kim et al., 2008; King et al., 2015). In this study, 23.3% of the isolates were A-B+CDT-, which is in agreement with previous studies in China in which the average prevalence rate of A-B+ strains is 21.4% (201/937), ranging from 0 to 53.6% by geographic distribution (Huang et al., 2009, 2010; Dong et al., 2013; Hawkey et al., 2013; Yan et al., 2013; Chen et al., 2014; Fang et al., 2014; Wang et al., 2014; Zhou et al., 2014; Cheng et al., 2015; Ye et al., 2015; Tian et al., 2016). Interestingly, infection with CDT-positive *C. difficile* strains has been reported to be associated with higher mortality and recurrence rates (Stewart et al., 2013). In this study, 5.1% (6/116) isolates were A+B+CDT+, which is slightly higher than in previous studies in Shanghai (1.6%, 2/110) and Zhejiang provinces (0%, 0/82; Huang et al., 2010; Fang et al., 2014). However, the overall prevalence of A+B+CDT+ *C. difficile* strains in China is much lower than in North America and Europe (Bassetti et al., 2012), possibly due to low lab diagnostic capacity and subsequent lack of awareness amongst medical personnel. By *tcdC* sequencing, five of six A+B+CDT+ isolates detected in this study had an 18-bp deletion at nucleotides 330–347, but only two ribotype 027 isolates, characterized by a single deletion at nucleotide 117, were detected.

The predominant ribotype and ST identified in the present study was ribotype 012/ST54 (16.4%), followed by ribotypes 001/ST3 (14.7%) and 017/ST37 (13.8%), whilst the commonest ribotype is ribotype 012 reported overall in China (100 of 589 isolates, 17.0%), and ST54, ST3, ST35, and ST37 (prevalence of 21.4, 12.6, 11.0, and 9.4 of 682 isolates tested, respectively) rank the top four STs (Huang et al., 2009, 2010; Dong et al., 2013; Hawkey et al., 2013; Yan et al., 2013; Chen et al., 2014; Fang et al., 2014; Wang et al., 2014; Zhou et al., 2014; Cheng et al., 2015; Ye et al., 2015; Tian et al., 2016). The remaining ribotypes/STs were distributed sporadically among different departments and with a lower prevalence. Generally, there was no obvious correlation between clonal clusters of the organism generated from the different departments during the study period, suggesting that a wide variety of strains are implicated in this hospital. However, due to lack of consistent data and limited lab diagnostic capacity for CDI at the hospital concerned, these findings may be biased.

Although the major molecular types identified in different *C. difficile* studies were similar, the prevalence of different molecular types varied amongst studies in China. In agreement to our findings, ribotype 017 *C. difficile* strains have been reported in several Asian countries (Collins et al., 2013). However, in North America and Europe, many CDI cases have been attributed to *C. difficile* ribotype 027 and 078 strains which have been described in many hospitals, and were particularly associated with outbreaks or epidemics (Bassetti et al., 2012). The major clone implicated in epidemics varies according to geographic locale, highlighting the significance of establishing a local surveillance network for CDI.

The emergence of increased antibiotic resistance in different *C. difficile* strain types worldwide may be a contributing factor to increased occurrence of CDI outbreaks in several hospitals. Although several studies have reported on the increasing MIC for metronidazole and vancomycin in *C. difficile* (Baines et al., 2008; Adler et al., 2015), both drugs showed high *in vitro* activity against all the isolates in our study. Furthermore, all strains were also susceptible to meropenem and piperacillin/tazobactam. Although a small number (12.9%) of *C. difficile* strains with high-level resistance to rifampin and rifaximin was observed in the present study, the rate was lower than the 25–29% reported in Shanghai, China (Huang et al., 2009, 2010).

The high rate of drug resistance for ciprofloxacin (100%), erythromycin (69%), and clindamycin (87.9%) among the studied isolates suggests that use of these antibiotics may be a risk factor for the development of CDI in this geographic locale (Bassetti et al., 2012). In addition, A-B+ strains in the present study exhibited higher drug resistance rates compared to A+B+ strains for the antibiotics clindamycin, erythromycin, levofloxacin, rifampicin, rifaximin, and tetracycline. Thus toxigenic typing may be valuable for understanding antibiotic resistance in the management and control of CDI. Furthermore, the drug resistance rates of strains of different PCR ribotypes differed. These findings emphasize the importance of implementing active surveillance and molecular epidemiologic studies on CDI.

All the typing methods used in this study have the advantage of high reproducibility (**Table 4**). MLVA exhibited extremely high discriminatory power, and thus may be a potential valuable tool for investigating CDI outbreaks (Eyre et al., 2013b). However, a reasonable cut-off value is needed since *C. difficile* isolates from the same outbreak may belong to genetically closely related but different MLVA types (**Table 4**) (Marsh et al., 2006).

**Table 4:** Comparison and suggested usage of typing methods employed in the present study.

Typing method	Reproducibility	Inter-laboratory exchange	Discriminatory power	Running costs	Suggested usage
Toxigenic typing	+++	++	+	+	Toxin status confirmation and risk assessment for virulence
*tcdC* genotyping	+++	+	++	+	Hypervirulence strain detection
*slpA* genotyping	+++	+	++	+	Regional epidemiology for vaccine development
MLST	+++	+++	++	+++	Epidemiology surveillance (high costs); phylogenetic studies
Ribotyping	+++	+	++	+	Epidemiology surveillance (low costs); infection prevention and control
MLVA	+++	++	+++	++	Detection of local outbreaks

Based on our findings, we recommend PCR ribotyping as the method of choice for epidemiological surveillance and infection prevention and control, as it delivers high discrimination, accuracy, and reproducibility. However, more work is needed for its standardization and database construction (**Table 4**) (Indra et al., 2008; Fawley et al., 2015). Although MLST also offers high discriminatory power index and easy inter-laboratory data comparison which may be useful for epidemiology surveillance and phylogenetic studies, the high cost is prohibitive (**Table 4**) (Killgore et al., 2008; Collins et al., 2015; Tian et al., 2016).

Sequencing of the *slpA* gene is valuable for comparison of *C. difficile* strains implicated in epidemics in diverse areas because the typing results are reproducible and can easily be shared. In addition, as the *slpA* gene is related to the strain’s serogroup, this typing method could be useful in vaccine development (Kato et al., 2005; Tian et al., 2016). *tcdC* genotyping had the lowest *D*-value, but sequencing of this gene may be used as an indicator for the hyper-virulent *C. difficile* clones, e.g., PCR ribotype 027/078 isolates (Curry et al., 2007).

Several other *C. difficile* typing methods apart from the ones applied in this study have been used, including PFGE, REA, and PCR-restriction fragment length polymorphism (RFLP). Unfortunately, these restriction enzyme-agarose gel electrophoresis-based methods have poor reproducibility, and are difficult to standardize for result interpretation and inter-laboratory data exchange (Killgore et al., 2008; Collins et al., 2015). Recent advances in whole-genome sequencing of *C. difficile* has the potential to provide even greater epidemiological information, but is still premature for use in large scale epidemiologic studies (Eyre et al., 2013a; Knight et al., 2015).

## Conclusion

Our study is the most systematic study to integrate molecular epidemiology and antibiotic susceptibility testing, and provides comprehensive data for a better understanding of CDI in China. The findings highlight the importance of active surveillance using molecular typing techniques for better management and control of CDI in developing countries. Different molecular typing assays could be used depending on the scale and aim of the surveillance.

## Author Contributions

J-WC, MX, TK, and FK wrote the manuscript; Z-PX, LZ, and XF collaborated in molecular investigations of the strains; L-YS and X-LX summarized the patient’s medical records; Y-CX designed and supervised the study.

## Conflict of Interest Statement

The authors declare that the research was conducted in the absence of any commercial or financial relationships that could be construed as a potential conflict of interest.

## Footnotes

1 https://webribo.ages.at/

2 http://pubmlst.org/cdifficile/
